# Relationship of Soluble RAGE with Insulin Resistance and Beta Cell Function during Development of Type 2 Diabetes Mellitus

**DOI:** 10.1155/2015/150325

**Published:** 2015-05-19

**Authors:** Subrata Kumar Biswas, Sabreena Mohtarin, Sonchita Rani Mudi, Taznuva Anwar, Laila Anjuman Banu, Sheikh Md. Khorshed Alam, Md. Fariduddin, M. Iqbal Arslan

**Affiliations:** ^1^Department of Biochemistry, Bangabandhu Sheikh Mujib Medical University (BSMMU), Shahbag, Dhaka 1000, Bangladesh; ^2^Department of Biochemistry and Cell Biology, Bangladesh Institute of Research and Rehabilitation in Diabetes, Endocrine and Metabolic Disorders (BIRDEM), 122 Kazi Nazrul Islam Avenue, Shahbag, Dhaka 1000, Bangladesh; ^3^Department of Anatomy, Bangabandhu Sheikh Mujib Medical University (BSMMU), Shahbag, Dhaka 1000, Bangladesh; ^4^Department of Endocrinology, Bangabandhu Sheikh Mujib Medical University (BSMMU), Shahbag, Dhaka 1000, Bangladesh

## Abstract

This study examined whether circulating levels of soluble receptor for advanced glycation end products (sRAGE) alter in prediabetes and correlate with insulin resistance (IR) and beta cell function in prediabetes and newly diagnosed type 2 diabetes mellitus (T2DM). Subjects without previous history of diabetes were recruited and grouped as control, prediabetes, and newly diagnosed T2DM. The control subjects (*n* = 40) and people with prediabetes (*n* = 52) and diabetes (*n* = 66) were similar in terms of age, sex, BMI, systolic and diastolic BP, and fasting insulin level. HOMA-IR was found significantly higher in people with diabetes than control subjects (*p* < 0.001) and people with prediabetes (*p* = 0.005); and HOMA-%B was found significantly deteriorated in people with diabetes (*p* < 0.001) compared to control subjects and people with prediabetes. However, serum sRAGE levels did not show any significant alteration in people with prediabetes compared to control subjects. Moreover, univariate and multivariate analyses did not identify any significant correlation and statistical association of sRAGE with HOMA-IR and HOMA-%B in people with prediabetes and newly diagnosed T2DM. Our data suggest that serum sRAGE levels do not alter in people with prediabetes compared to control subjects and do not correlate or associate with IR and beta cell function during development of T2DM.

## 1. Introduction

The receptor for advanced glycation end products (RAGE) is a cell surface receptor of immunoglobulin superfamily. RAGE activation through ligand binding can induce chronic inflammation and oxidative stress, and it has been linked with diseases like diabetic complications, cardiovascular and neurodegenerative diseases, and cancer [1]. A soluble form of RAGE (sRAGE), which is a splice variant of full-length RAGE or a shedding/cleavage product of membrane-bound RAGE, has been found circulating in the plasma [2, 3]. The sRAGE can bind and sequester RAGE ligands and thereby can reduce RAGE activation. Therefore, the sRAGE is generally considered as protective against diseases originating from RAGE activation [1–3].

RAGE-ligand interaction was previously claimed to be involved in the pathogenesis of autoimmune diabetes, and treatment with sRAGE was shown to effectively prevent transfer of diabetes into NOD/*scid* mice that receive spleen cells from a diabetic NOD donor [4]. Subsequently, blockade of high-mobility group box 1, a RAGE ligand, was shown to inhibit insulitis progression and diabetes development in NOD mice [5]. A minor role of genetic variation in RAGE was also suggested to be associated with insulin resistance (IR) in a human population [6]. However, recent studies have suggested that low levels of circulating sRAGE may be involved in the development of diabetes mellitus [7–9]. A declining level of sRAGE at the time of seroconversion to autoantibody positivity has been suspected to be a predictor of type 1 diabetes [7, 8], and an independent association has been found between low levels of sRAGE and development of type 2 diabetes mellitus (T2DM) [9]. However, the relationship of sRAGE with the underlying pathophysiological mechanisms of T2DM has not been specifically explored.

The IR and beta cell dysfunction are two core defects of T2DM, and the “prediabetes” is a category of increased risk of developing T2DM in subjects who have not yet fulfilled the criteria to be diabetic [10]. To explore the involvement or participation of sRAGE in the development of T2DM, present study was designed to assess whether sRAGE levels alter in prediabetes and correlate with IR and beta cell function in prediabetes and newly diagnosed T2DM.

## 2. Subjects and Methods 

A total of 158 participants were recruited from those who came for diabetes screening at the Bangabandhu Sheikh Mujib Medical University, Dhaka, Bangladesh, after giving written consent. This cross-sectional study was conducted according to the Declaration of Helsinki and was approved by the institutional ethical review committee. Participants were grouped as control (normoglycemic), prediabetes, and newly diagnosed T2DM based on their blood glucose (fasting and 2 hrs after 75 grams glucose load) and HbA1c levels. As recommended by American Diabetic Association (ADA) [10], diabetes was considered with a fasting glucose level ≥7.0 mmol/L and/or 2 hrs blood glucose ≥11.1 mmol/L and/or HbA1c ≥6.5%; and prediabetes was considered with a fasting glucose level 5.6–6.9 mmol/L (impaired fasting glucose, IFG) and/or 2 hrs blood glucose 7.8–11.0 mmol/L (impaired glucose tolerance, IGT) and/or HbA1c 5.7–6.4%. Control subjects did not qualify for any of the above glycemic or HbA1c criteria. Subjects with previous history of diabetes and those suffering from hypertension, chronic liver and kidney diseases, or any other acute/chronic inflammatory conditions as well as pregnant and lactating women and regular drug users were excluded. A detailed medical history was taken and clinical examination including height, weight, and blood pressure data were recorded for all subjects.

A fasting blood sample was collected after an overnight fasting of >8 hours and a second blood sample was collected 2 hours after 75 grams glucose load on the same day from all subjects. Fasting and 2 hours after glucose levels were measured by enzymatic spectrophotometric method using Dimension RxL Max clinical chemistry analyzer (Siemens Healthcare Diagnostics Inc., Newark, DE, USA). HbA1c levels were measured by ion-exchange high-performance liquid chromatography in a Bio-Rad D-10 instrument (Bio-Rad Laboratories Inc., Hercules, CA, USA). Fasting serum insulin levels were measured by microparticle enzyme immunoassay technique (Abbott Diagnostics, Wiesbaden, Germany) using an Abbott AxSYM system with an interassay coefficient of variation (CV) < 5%. Fasting serum sRAGE levels were measured by ELISA in triplicate, as suggested by the manufacturer (R&D Systems, Minneapolis, MN, USA) with an interassay CV of <7%. The IR and beta cell function were calculated as homeostasis model assessment of IR (HOMA-IR) [(glucose × insulin)/22.5] and homeostasis model assessment of beta cell function (HOMA-%B) [(20 × insulin)/(glucose − 3.5)], respectively, where glucose was in mmol/L and insulin was in *µ*U/mL.

*Statistical Analysis*. All statistical analyses were performed using SPSS version 20.0 (SPSS Inc., Chicago, IL). Data are presented as mean ± SD. Variables with a skewed distribution are expressed as median (interquartile range) and were log transformed before statistical analysis. Comparison among multiple groups was done by one-way ANOVA followed by Bonferroni corrected* t*-test. Categorical variables were analyzed by *χ* ^2^ test. Bivariate correlations were determined by Pearson's univariate correlation analysis. Stepwise multivariate linear regression models were calculated to demonstrate independent relationships of sRAGE and other variables with HOMA-IR and HOMA-%B. The HOMA-IR was used as dependent variable in one model and the HOMA-%B as dependent variable in another model with the following independent variables: age, sex, BMI, systolic and diastolic BP, glucose 2 hours, HbA1c, sRAGE, and HOMA-IR/HOMA-%B. A *p* value of ≤0.05 for *F*-values was taken as criterion for entering variables in the model and *p* ≥ 0.1 for *F*-values was taken as criterion for exclusion of variables from the model. Fasting glucose and insulin levels were not included in the models since they were directly used for calculation of HOMA-IR and HOMA-%B. A two-tailed value of *p* < 0.05 was considered statistically significant.

## 3. Results

As shown in Table 1, the age (40.2 ± 8.7; 20–58 years) and sex (m = 73, f = 85) distributions of the 158 study participants were found similar among control subjects (*n* = 40) and people with prediabetes (*n* = 52) and diabetes (*n* = 66). The BMI, systolic and diastolic BP, and fasting insulin levels were also found similar among the three groups. But the fasting and 2 hours blood glucose and HbA1c levels were found significantly (*p* < 0.001) elevated in people with diabetes compared to control subjects and people with prediabetes. HOMA-IR was found significantly higher in people with diabetes than control subjects (*p* < 0.001) and people with prediabetes (*p* = 0.005). As expected, HOMA-%B was found markedly decreased in people with diabetes (*p* < 0.001) compared to control subjects and people with prediabetes.

**Figure 1:**
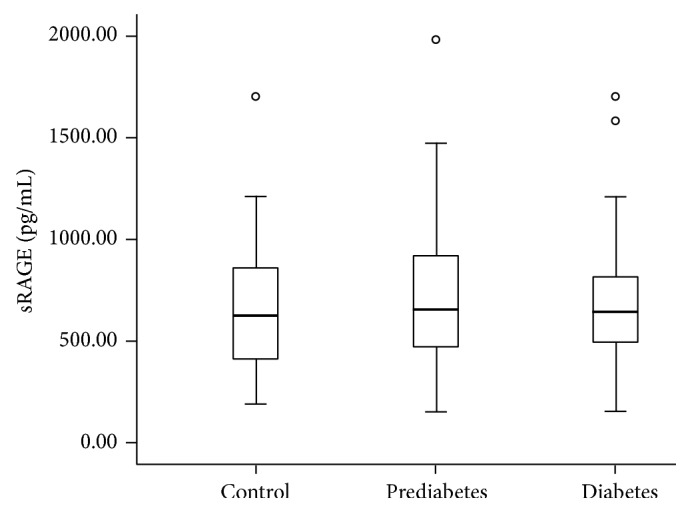
Box plot showing the sRAGE concentrations in control subjects and in people with prediabetes and newly diagnosed type 2 diabetes. The line within the boxes represents the median, the bottom of each box represents the 25th percentile, and the top of the box represents the 75th percentile. The whiskers represent the 5th and 95th percentiles, and the small circles represent outliers.

However, as shown in Figure 1, serum sRAGE levels in people with prediabetes (656, 463–968; median, interquartile range in pg/mL) did not show any significant difference compared with that of control subjects (626, 413–864) and people with diabetes (646, 493–817).

We next investigated the relationship of sRAGE with markers of IR and beta cell function by using Pearson's correlation test. The sRAGE level did not show any significant correlation with HOMA-IR in all the study subjects (*r* = 0.007, *p* = 0.94), in people with diabetes (*r* = −0.07, *p* = 0.64) or prediabetes (*r* = 0.22, *p* = 0.17). Similarly, sRAGE level did not show any significant correlation with HOMA-%B in all the study subjects (*r* = −0.02, *p* = 0.87), in people with diabetes (*r* = 0.04, *p* = 0.80) or prediabetes (*r* = 0.24, *p* = 0.12) (data not shown in the table). For further statistical analysis, we merged the people with prediabetes and newly diagnosed type 2 diabetes together (PD + DM group, *n* = 118) considering that both groups have similar underlying pathophysiological defects responsible for glucose intolerance and hyperglycemia. Characteristics of the participants of this PD + DM group as well as relationship of HOMA-IR and HOMA-%B with other variables were shown in Table 2. Of note, sRAGE levels did not show any significant correlation with HOMA-IR and HOMA-%B even in the participants of this PD + DM group (Table 2). But HOMA-IR showed marginal correlation with 2 hours glucose levels (*r* = 0.20, *p* = 0.05) and significant correlation with HOMA-%B (*r* = 0.27, *p* = 0.007), and HOMA-%B showed significant correlation with 2 hours glucose levels (*r* = 0.70, *p* < 0.001) and HbA1c (*r* = 0.72, *p* < 0.001) (Table 2).

A stepwise multivariate linear regression model with HOMA-IR as dependent variable and age, sex, BMI, systolic and diastolic BP, 2 hours glucose level, HbA1c, sRAGE, and HOMA-%B as independent variables showed independent association of HOMA-IR with BMI (*β* = 0.21, *p* = 0.03), 2 hours glucose levels (*β* = 0.67, *p* < 0.001), and HOMA-%B (*β* = 0.74, *p* < 0.001) in PD + DM group (*R* ^2^ = 0.405) (Table 2).

Another model with HOMA-%B as dependent variable and age, sex, BMI, systolic and diastolic BP, 2 hours glucose level, HbA1c, sRAGE, and HOMA-IR as independent variables showed independent association of HOMA-%B with 2 hours glucose level (*β* = 0.33, *p* = 0.028), HbA1c (*β* = 0.41, *p* = 0.005), and HOMA-IR (*β* = 0.46, *p* < 0.001) in PD + DM group (*R* ^2^ = 0.629) (Table 2). Furthermore, the above regression models when applied separately for the people with prediabetes (not shown in the table) showed significant association of HOMA-IR with BMI (*β* = 0.33, *p* = 0.006) and HOMA-%B (*β* = 0.62, *p* < 0.001) (*R* ^2^ = 0.530) and HOMA-%B only with HOMA-IR (*β* = 0.67, *p* < 0.001, *R* ^2^ = 0.433). Such models when applied for the people with diabetes (not shown in the table) showed significant association of HOMA-IR with HbA1c (*β* = 0.43, *p* = 0.009) and HOMA-%B (*β* = 0.92, *p* < 0.001) (*R* ^2^ = 0.536) and HOMA-%B with HbA1c (*β* = −0.52, *p* < 0.001) and HOMA-IR (*β* = 0.61, *p* < 0.001) (*R* ^2^ = 0.694). But none of the above models showed any significant association of sRAGE with HOMA-IR and HOMA-%B in people with prediabetes and newly diagnosed T2DM.

## 4. Discussion

The T2DM develops insidiously with gradual impairment of glucose tolerance due to IR and beta cell dysfunction [10]. Before development of overt diabetes mellitus there is a state of prediabetes characterized by IGT and/or IFG. People with prediabetes suffer from increased risk of developing T2DM in near future compared with people with normal glucose tolerance [10]. In the present study we investigated the sRAGE levels in prediabetes and the relationship of sRAGE with IR and beta cell function in people with prediabetes and newly diagnosed T2DM. We found that sRAGE levels do not alter in people with prediabetes compared with normoglycemic control subjects. Moreover, we did not observe any correlation or statistical association of sRAGE with IR and beta cell function in people with prediabetes and newly diagnosed T2DM.

Discrepancy exists regarding the sRAGE levels in diabetes—higher, lower, and even similar levels of sRAGE have been reported in people with T2DM compared with control subjects without diabetes (reviewed in [11]). The reason for this discrepancy among studies is not clear but the presence of confounding variables like the duration of diabetes, presence of hypertension and use of antihypertensive drugs, smoking habit, and chronic kidney and inflammatory diseases may contribute to this [11, 12]. It has been shown that longer duration of diabetes is associated with increased advanced glycation end products (AGE) generation and AGE-stimulated increased RAGE expression. The increased RAGE expression in turn may increase sRAGE level by shedding of membrane-bound RAGE [11]. If so, people with prediabetes and newly-diagnosed T2DM may not show a significant alteration in sRAGE level since they may not have experienced a heavy load of AGE yet. In fact, a large study recently found no difference in sRAGE levels between children with newly diagnosed type 1 diabetes and control subjects and emphasized the importance of evaluating sRAGE levels in children with prediabetes [13]. But, to our knowledge, the sRAGE status in people with prediabetes was unknown until recently. During the preparation of this paper, Di Pino et al. published cardiovascular risk profile in prediabetes and type 2 diabetes, where they found similar levels of sRAGE in control subjects and in people with prediabetes and new-onset T2DM [14] as we found in the present study. However, it should be noted that 68% of the people with prediabetes of the study by Di Pino et al. were normal glucose tolerant (without having IFG or IGT) since Di Pino et al. grouped the study subjects only on the basis of HbA1c. Thus it was uncertain whether the findings of Di Pino et al. would be equally valid for people with prediabetes defined by standard ADA criteria (IFG and/or IGT and/or HbA1c 5.7–6.4%) [10]. In the present study, we significantly added to the findings of Di Pino et al. by showing that sRAGE levels do not alter in people with prediabetes, defined by standard ADA criteria, compared with control subjects and people with newly diagnosed T2DM.

The IR and beta cell dysfunction are two core defects that are found in variable extent in people with T2DM [10]. However, the relationship of sRAGE with IR and beta cell function was so far not clear. To our knowledge, Basta et al. previously found negative correlation between sRAGE level and IR taken control subjects and people with diabetes together in the analysis [15]. But this relationship disappeared when they analyzed the data in age-selected control subjects and people with diabetes separately. Furthermore, an independent negative association of sRAGE with IR also disappeared when they performed multivariate regression analyses on control subjects and people with diabetes separately [15]. In fact, the people with diabetes of the study by Basta et al. [15] were significantly different from control subjects in respect to age, number of hypertensive subjects, and use of antihypertensive drugs, factors that are known to affect sRAGE levels [11]. Taken together, the negative relationship between sRAGE and IR shown by Basta et al. was questionable. In the present study in a relatively homogenous set of study subjects we found that sRAGE levels do not correlate and do not show any association with IR in people with prediabetes and newly diagnosed T2DM. Furthermore we found for the first time that sRAGE levels do not correlate and do not show any association with beta cell function in people with prediabetes and newly diagnosed T2DM.

The global prevalence of diabetes mellitus is rapidly rising and it is generally considered that sedentary but stressful life-style along with unhealthy food habits and other environmental factors may be responsible for this. It has been shown that dietary factors may contribute to excess accumulation of AGEs in the body [16], and AGEs have been suggested to promote beta cell dysfunction [17, 18] and dietary restriction of AGEs has been reported to reduce the incidence of diabetes in a mouse model of autoimmune diabetes [19]. Moreover, AGEs can act through RAGE activation, and the exogenous sRAGE and other inhibitors of RAGE ligand were shown to inhibit the development of diabetes in NOD mice [4, 5]. Recently several studies have shown a decrease in circulating concentrations of sRAGE at the time of seroconversion to autoantibody positivity in children with prediabetes before development of type 1 diabetes [7, 8]. These authors proposed that a declining level of sRAGE with simultaneous decrease in sRAGE/AGE ratio at seroconversion may represent a failing protection of beta cells against harmful AGEs since sRAGE can bind excessive AGEs [8]. At the same time, low circulating sRAGE at baseline has recently been shown to be significantly and independently associated with future risk of T2DM, coronary heart disease, and all-cause mortality during a median of 18 years of follow-up in a community-based population [9]. However, this latter study was criticized as previous studies had shown higher, but not lower, levels of sRAGE are independently associated with the risk of future cardiovascular disease and all-cause mortality [11, 20–22]. Moreover, circulating sRAGE levels were shown to be 1,000 times lower than needed to be efficiently capturing the circulating AGEs and therefore it is unlikely that the low levels of endogenous sRAGE to counteract the detrimental effect of AGEs might be involved in the future risk of T2DM or cardiovascular disease [11, 22]. Our present finding of no relationship of endogenous sRAGE with IR and beta cell function also supports this explanation.

It was previously uncertain whether sRAGE levels alter in prediabetes and whether sRAGE levels hold any relationship with the underlying core defects of diabetes during development of T2DM, which in the present study we have tried to explore. However, we are fully aware of the limited sample size and the cross-sectional nature of our study. Future studies are therefore required to prospectively and serially measure sRAGE levels in the same subjects who develop T2DM from normoglycemia through prediabetes and to compare sRAGE with the evolution of IR and beta cell dysfunction in those individuals.

In summary, we concluded that sRAGE levels do not change in prediabetes and do not show any relationship with IR and beta cell function in people with prediabetes and newly diagnosed T2DM. These findings suggest that sRAGE is unlikely to be an important predictor of insulin resistance and beta cell dysfunction during development of T2DM. However, further studies are needed to explore the dynamics of sRAGE during development of T2DM.

## Figures and Tables

**Table 1:** Clinical and biochemical characteristics of the study subjects.

Variables	Control subjects (*n* = 40)	People with prediabetes (*n* = 52)	People with diabetes (*n* = 66)
Age (years)	38.4 ± 7.6	38.9 ± 9.5	42.5 ± 8.4
Sex, male/female (*n*)	18/22	24/28	31/35
BMI (kg/m^2^)	25.9 ± 6.2	27.2 ± 5.5	25.4 ± 4.6
SBP (mm Hg)	116.3 ± 8.5	118.1 ± 8.6	119.3 ± 8.3
DBP (mm Hg)	74.7 ± 8.4	76.1 ± 7.8	75.4 ± 8.2
Glucose, fasting (mmol/L)	4.7 ± 0.5	5.1 ± 0.6	9.7 ± 3.6^a^
Glucose, 2 hrs (mmol/L)	6.5 ± 0.9	8.2 ± 1.4	16.3 ± 5.6^a^
HbA1c (%)	5.0 ± 0.4	5.7 ± 0.5	8.1 ± 2.4^a^
HbA1c (mmol/mol)	30.9 ± 4.3	38.4 ± 5.4	65.5 ± 26.0^a^
Insulin^∗^ (*µ*U/mL)	9.3 (6.7–14.8)	11.5 (8.6–17.8)	9.4 (5.9–18.6)
HOMA-IR^∗^	2.0 (1.6–3.1)	2.6 (1.9–4.1)	4.1 (2.7–7.7)^bc^
HOMA-%B^∗^ (%)	168 (95–313)	149 (109–230)	39 (19–89)^a^

Data are mean ± SD, number, and median (interquartile range). ^∗^Log-transformed variables, values given are median (interquartile range). ^a^ *p* < 0.001 versus control subjects and people with prediabetes; ^b^ *p* < 0.001 versus control subjects; ^c^ *p* = 0.005 versus people with prediabetes. BMI, body mass index; SBP, systolic blood pressure; DBP, diastolic blood pressure; HOMA-IR, homeostasis model assessment of insulin resistance; HOMA-%B, homeostasis model assessment of beta cell function.

**Table 2:** Patient characteristics and associations of HOMA-IR and HOMA-%B with different variables in people with prediabetes and newly diagnosed type 2 diabetes.

	Characteristics PD + DM (*n* = 118)	HOMA-IR^∗^		HOMA-%B^∗^	
		Univariate (*r*)	Multivariate (*β*)	Univariate (*r*)	Multivariate (*β*)
Age (years)	40.8 ± 9.0	0.06	—	−0.06	—
Sex, m/f (*n*)	55/63	—	—	—	—
BMI (Kg/m^2^)	26.17 ± 5.03	0.12	**0.21** ^a^	0.19	—
SBP (mm Hg)	118.8 ± 8.4	−0.03	—	−0.12	—
DBP (mm Hg)	75.7 ± 8.0	−0.09	—	−0.13	—
Glucose, 2 hrs (mmol/L)	12.8 ± 5.9	**0.20** ^a^	**0.67** ^c^	**−0.70** ^c^	**−0.33** ^a^
HbA1c (%)	6.9 ± 2.1	0.06	—	**−0.72** ^c^	**−0.41** ^b^
HOMA-IR^∗^	3.2 (2.3–5.3)	—	—	**0.27** ^b^	**0.46** ^c^
HOMA-%B^∗^	95 (33–163)	**0.27** ^b^	**0.74** ^c^	—	—
sRAGE (pg/mL)^∗^	646 (482–897)	0.04	—	0.08	—

Data are mean ± SD, number, and median (interquartile range). ^∗^Values were not normally distributed and log-transformed for statistical analysis. *r*, Pearson's univariate correlation coefficient; *β*, standardized coefficient as given by stepwise multivariate regression analysis. Dashes in the multivariate columns correspond to variables excluded from the model due to *p* ≥ 0.1 for *F*-values. PD + DM, people with prediabetes and diabetes together; BMI, body mass index; SBP, systolic blood pressure; DBP, diastolic blood pressure; ^a^ *p* ≤ 0.05; ^b^ *p* < 0.01; ^c^ *p* < 0.001.

## Abbreviations

AGE: Advanced glycation end products
HOMA-IR: Homeostasis model assessment of insulin resistance
HOMA-%B: Homeostasis model assessment of beta cell function
RAGE: Receptor for advanced glycation end products
sRAGE: Soluble receptor for advanced glycation end products
T2DM: Type 2 diabetes mellitus
PD + DM: Prediabetes and diabetes mellitus together.
